# *N*-[2-(2-Chloro­phen­yl)-2-hydroxy­ethyl]propan-2-aminium nitrate

**DOI:** 10.1107/S1600536809054506

**Published:** 2010-01-16

**Authors:** Hai Feng, Zhan Tang, Lin-Jun Xie, Bin-Tao Xing

**Affiliations:** a College of Pharmaceutical Sciences, Zhejiang University of Technology, Hangzhou 310014, People’s Republic of China; b College of Mechanical Engineering, Zhejiang University of Technology, Hangzhou 310014, People’s Republic of China

## Abstract

In the title compound, C_11_H_17_ClNO^+^·NO_3_^−^, the side chain of the ethyl­ammonium group is orientated approximately perpendicular to the benzene ring, the dihedral angle between the C/C/N plane of the ethyl­ammonium group and the benzene ring being 79.40 (18)°. In the crystal structure, inter­molecular O—H⋯O and N—H⋯O hydrogen bonds are observed between the cation and the anion.

## Related literature

For related structures, see: Tang, Xu, Zhang & Feng (2009 ▶); Tang, Xu, Zheng & Feng (2009 ▶).
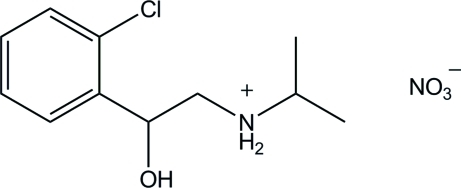


## Experimental

### 

#### Crystal data

C_11_H_17_ClNO^+^·NO_3_^−^
*M* *_r_* = 276.72
Monoclinic, 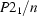

*a* = 11.9551 (6) Å
*b* = 10.4563 (5) Å
*c* = 12.2968 (7) Å
β = 115.109 (1)°
*V* = 1391.91 (12) Å^3^
*Z* = 4
Mo *K*α radiation
μ = 0.28 mm^−1^
*T* = 296 K
0.38 × 0.36 × 0.22 mm

#### Data collection

Rigaku R-AXIS RAPID diffractometer
Absorption correction: multi-scan (*ABSCOR*; Higashi, 1995 ▶) *T*_min_ = 0.900, *T*_max_ = 0.940
13380 measured reflections
3179 independent reflections
1833 reflections with *I* > 2σ(*I*)
*R*_int_ = 0.031

#### Refinement

*R*[*F* ^2^ > 2σ(*F* ^2^)] = 0.045
*wR*(*F* ^2^) = 0.145
*S* = 1.00
3179 reflections
167 parameters
H-atom parameters constrained
Δρ_max_ = 0.37 e Å^−3^
Δρ_min_ = −0.49 e Å^−3^

### 

Data collection: *PROCESS-AUTO* (Rigaku/MSC, 2006 ▶); cell refinement: *PROCESS-AUTO*; data reduction: *CrystalStructure* (Rigaku/MSC, 2007 ▶); program(s) used to solve structure: *SHELXS97* (Sheldrick, 2008 ▶); program(s) used to refine structure: *SHELXL97* (Sheldrick, 2008 ▶); molecular graphics: *ORTEP-3 for Windows* (Farrugia, 1997 ▶); software used to prepare material for publication: *WinGX* (Farrugia, 1999 ▶).

## Supplementary Material

Crystal structure: contains datablocks global, I. DOI: 10.1107/S1600536809054506/is2504sup1.cif

Structure factors: contains datablocks I. DOI: 10.1107/S1600536809054506/is2504Isup2.hkl

Additional supplementary materials:  crystallographic information; 3D view; checkCIF report

## Figures and Tables

**Table 1:** Hydrogen-bond geometry (Å, °)

*D*—H⋯*A*	*D*—H	H⋯*A*	*D*⋯*A*	*D*—H⋯*A*
N1—H2*A*⋯O4	0.90	1.97	2.843 (2)	163
N1—H2*B*⋯O2^i^	0.90	1.93	2.8234 (19)	170
O1—H101⋯O4^ii^	0.82	1.98	2.7614 (19)	158

Symmetry codes: (i) 
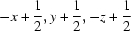
; (ii) 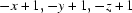.

**Fig. 1.**
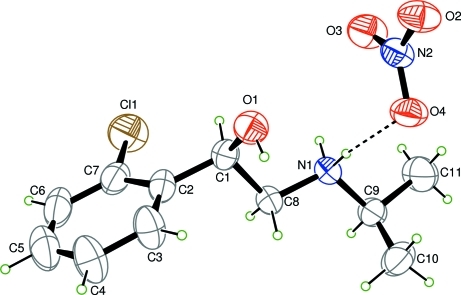
The molecular structure of the title compound, with atom labels, showing 40% probability displacement ellipsoids.

## supplementary crystallographic information

### Comment

A recent study reports the structure of bis{*N*-[2-(2-chlorophenyl)-2-hydroxyethyl]propan-2-aminium} oxalate (Tang, Xu, Zhang & Feng, 2009), which was synthesized by oxalic acid and clorprenaline (Tang, Xu, Zheng & Feng, 2009). Here using nitric acid instead of oxalic acid and following a similar synthetic procedure yields the title compound, (I).

In the molecular structure (Fig. 1), the Cl atom and the phenyl plane is almost planar with a deviation of 0.0118 Å. The dihedral angle between the plane formed by C1/C2/C8 and the benzene plane is 81.23 (18)°, which shows that the two planes are almost perpendicular. O—H···O and N—H···O hydrogen bonds are found in the crystal structure.

### Experimental

Racemic clorprenaline was prepared by clorprenaline hydrochloride purchased from ShangHai Shengxin Medicine & Chemical Co., Ltd. ShangHai, China. Clorprenaline hydrochloride and NaOH in a molar ratio of 1:1 were mixed and dissolved in a methanol-water solution (1:1 *v*/*v*). The precipitate formed was filtered off, washed with water and dried. It was used without further purification. Racemic clorprenaline (3.0 g, 0.014 mol) was dissolved in ethanol (30 ml), then nitric acid was added to give pH of about 2. The resulting solution was concentrated and colorless crystals of (I) were obtained within one day at ambient temperature.

### Refinement

All H atoms were placed in calculated positions and allowed to ride on their parent atoms, with C—H = 0.93 (aromatic), 0.98 (methine), 0.97 (methylene), 0.96 Å (methyl), O—H = 0.82 Å and N—H = 0.90 Å, and with *U*_iso_(H) = 1.2–1.5 times *U*_eq_ of the parent atoms.

### Crystal data

**Table:**

C_11_H_17_ClNO^+^·NO_3_^−^	*F*(000) = 584
*M**_r_* = 276.72	*D*_x_ = 1.320 Mg m^−^^3^
Monoclinic, *P*2_1_/*n*	Mo *K*α radiation, λ = 0.71073 Å
Hall symbol: -P 2yn	Cell parameters from 7750 reflections
*a* = 11.9551 (6) Å	θ = 3.1–27.4°
*b* = 10.4563 (5) Å	µ = 0.28 mm^−^^1^
*c* = 12.2968 (7) Å	*T* = 296 K
β = 115.109 (1)°	Chunk, colorless
*V* = 1391.91 (12) Å^3^	0.38 × 0.36 × 0.22 mm
*Z* = 4	

### Data collection

**Table:**

Rigaku R-AXIS RAPID diffractometer	3179 independent reflections
Radiation source: rolling anode	1833 reflections with *I* > 2σ(*I*)
graphite	*R*_int_ = 0.031
Detector resolution: 10.00 pixels mm^-1^	θ_max_ = 27.4°, θ_min_ = 3.1°
ω scans	*h* = −15→15
Absorption correction: multi-scan (*ABSCOR*; Higashi, 1995)	*k* = −13→13
*T*_min_ = 0.900, *T*_max_ = 0.940	*l* = −15→15
13380 measured reflections	

### Refinement

**Table:**

Refinement on *F*^2^	Secondary atom site location: difference Fourier map
Least-squares matrix: full	Hydrogen site location: inferred from neighbouring sites
*R*[*F*^2^ > 2σ(*F*^2^)] = 0.045	H-atom parameters constrained
*wR*(*F*^2^) = 0.145	*w* = 1/[σ^2^(*F*_o_^2^) + (0.0508*P*)^2^ + 0.8267*P*] where *P* = (*F*_o_^2^ + 2*F*_c_^2^)/3
*S* = 1.00	(Δ/σ)_max_ < 0.001
3179 reflections	Δρ_max_ = 0.37 e Å^−^^3^
167 parameters	Δρ_min_ = −0.49 e Å^−^^3^
0 restraints	Extinction correction: *SHELXL97* (Sheldrick, 2008), Fc^*^=kFc[1+0.001xFc^2^λ^3^/sin(2θ)]^-1/4^
Primary atom site location: structure-invariant direct methods	Extinction coefficient: 0.031 (2)

### Special details

**Table:**

Geometry. All e.s.d.'s (except the e.s.d. in the dihedral angle between two l.s. planes) are estimated using the full covariance matrix. The cell e.s.d.'s are taken into account individually in the estimation of e.s.d.'s in distances, angles and torsion angles; correlations between e.s.d.'s in cell parameters are only used when they are defined by crystal symmetry. An approximate (isotropic) treatment of cell e.s.d.'s is used for estimating e.s.d.'s involving l.s. planes.
Refinement. Refinement of *F*^2^ against ALL reflections. The weighted *R*-factor *wR* and goodness of fit *S* are based on *F*^2^, conventional *R*-factors *R* are based on *F*, with *F* set to zero for negative *F*^2^. The threshold expression of *F*^2^ > σ(*F*^2^) is used only for calculating *R*-factors(gt) *etc*. and is not relevant to the choice of reflections for refinement. *R*-factors based on *F*^2^ are statistically about twice as large as those based on *F*, and *R*- factors based on ALL data will be even larger.

### Fractional atomic coordinates and isotropic or equivalent isotropic displacement parameters (Å^2^)

**Table:**

	*x*	*y*	*z*	*U*_iso_*/*U*_eq_	
Cl1	0.50104 (7)	0.95849 (7)	0.13939 (6)	0.1022 (3)	
N2	0.28600 (14)	0.45390 (15)	0.33777 (14)	0.0580 (4)	
N1	0.37095 (12)	0.76881 (13)	0.40807 (13)	0.0468 (4)	
H2A	0.3748	0.6832	0.4169	0.056*	
H2B	0.3128	0.7862	0.3339	0.056*	
C8	0.49200 (14)	0.81542 (17)	0.41706 (16)	0.0489 (4)	
H8A	0.5552	0.7990	0.4973	0.059*	
H8B	0.4877	0.9071	0.4039	0.059*	
O1	0.53694 (12)	0.61603 (12)	0.34203 (12)	0.0634 (4)	
H101	0.5812	0.5995	0.4126	0.095*	
O2	0.28728 (13)	0.33459 (13)	0.33095 (13)	0.0724 (5)	
O3	0.23497 (15)	0.52046 (14)	0.24813 (13)	0.0787 (5)	
O4	0.33694 (14)	0.50469 (14)	0.43991 (12)	0.0734 (4)	
C9	0.33109 (16)	0.82528 (19)	0.49838 (17)	0.0568 (5)	
H8	0.3382	0.9186	0.4967	0.068*	
C1	0.52762 (16)	0.75053 (17)	0.32594 (16)	0.0521 (4)	
H1	0.4615	0.7673	0.2461	0.063*	
C2	0.64380 (17)	0.81197 (18)	0.33057 (17)	0.0560 (5)	
C7	0.6422 (2)	0.9062 (2)	0.25042 (18)	0.0650 (5)	
C3	0.75844 (18)	0.7782 (2)	0.4181 (2)	0.0776 (7)	
H3	0.7634	0.7155	0.4735	0.093*	
C10	0.4135 (2)	0.7788 (3)	0.62253 (19)	0.0801 (7)	
H9A	0.4150	0.6870	0.6232	0.096*	
H9B	0.3825	0.8089	0.6782	0.096*	
H9C	0.4957	0.8109	0.6455	0.096*	
C6	0.7495 (2)	0.9625 (2)	0.2564 (2)	0.0830 (6)	
H6	0.7457	1.0243	0.2006	0.100*	
C5	0.8610 (2)	0.9267 (3)	0.3447 (3)	0.0923 (7)	
H5	0.9333	0.9647	0.3496	0.111*	
C11	0.19628 (19)	0.7912 (3)	0.4610 (2)	0.0950 (9)	
H10A	0.1473	0.8255	0.3826	0.114*	
H10B	0.1690	0.8266	0.5176	0.114*	
H10C	0.1874	0.6999	0.4590	0.114*	
C4	0.8659 (2)	0.8349 (3)	0.4258 (3)	0.0942 (9)	
H4	0.9416	0.8106	0.4861	0.113*	

### Atomic displacement parameters (Å^2^)

**Table:**

	*U*^11^	*U*^22^	*U*^33^	*U*^12^	*U*^13^	*U*^23^
Cl1	0.1190 (5)	0.0985 (5)	0.0691 (3)	−0.0122 (4)	0.0207 (3)	0.0293 (3)
N2	0.0537 (8)	0.0524 (9)	0.0573 (9)	−0.0069 (7)	0.0134 (7)	0.0004 (7)
N1	0.0444 (7)	0.0437 (7)	0.0513 (7)	−0.0031 (6)	0.0193 (6)	−0.0005 (6)
C8	0.0448 (8)	0.0489 (9)	0.0546 (9)	−0.0036 (7)	0.0225 (7)	−0.0004 (7)
O1	0.0721 (8)	0.0509 (7)	0.0686 (8)	−0.0004 (6)	0.0312 (6)	−0.0032 (6)
O2	0.0794 (9)	0.0485 (7)	0.0677 (8)	0.0020 (7)	0.0105 (7)	−0.0035 (6)
O3	0.0865 (10)	0.0630 (8)	0.0633 (8)	−0.0017 (8)	0.0093 (7)	0.0125 (7)
O4	0.0934 (10)	0.0564 (8)	0.0567 (8)	−0.0187 (7)	0.0185 (7)	−0.0061 (6)
C9	0.0538 (9)	0.0598 (11)	0.0636 (10)	−0.0034 (8)	0.0315 (8)	−0.0127 (9)
C1	0.0557 (9)	0.0533 (10)	0.0497 (9)	0.0023 (8)	0.0247 (7)	0.0062 (8)
C2	0.0629 (9)	0.0551 (10)	0.0619 (10)	0.0007 (8)	0.0378 (8)	0.0024 (8)
C7	0.0842 (12)	0.0617 (12)	0.0610 (10)	−0.0040 (10)	0.0422 (9)	−0.0010 (9)
C3	0.0563 (10)	0.0833 (15)	0.0978 (15)	0.0062 (11)	0.0371 (10)	0.0233 (12)
C10	0.0859 (13)	0.1027 (18)	0.0609 (11)	−0.0001 (13)	0.0401 (10)	−0.0077 (11)
C6	0.1148 (14)	0.0703 (14)	0.0984 (13)	−0.0136 (13)	0.0785 (11)	−0.0028 (11)
C5	0.0847 (12)	0.0848 (16)	0.1380 (19)	−0.0117 (13)	0.0768 (13)	−0.0115 (15)
C11	0.0604 (11)	0.130 (2)	0.1072 (17)	−0.0126 (13)	0.0480 (11)	−0.0340 (16)
C4	0.0587 (11)	0.1000 (19)	0.132 (2)	0.0021 (13)	0.0478 (13)	0.0155 (16)

### Geometric parameters (Å, °)

**Table:**

Cl1—C7	1.748 (2)	C1—H1	0.9800
N2—O3	1.225 (2)	C2—C3	1.382 (3)
N2—O2	1.251 (2)	C2—C7	1.388 (3)
N2—O4	1.258 (2)	C7—C6	1.385 (3)
N1—C8	1.486 (2)	C3—C4	1.381 (3)
N1—C9	1.503 (2)	C3—H3	0.9300
N1—H2A	0.9000	C10—H9A	0.9600
N1—H2B	0.9000	C10—H9B	0.9600
C8—C1	1.517 (3)	C10—H9C	0.9600
C8—H8A	0.9700	C6—C5	1.366 (3)
C8—H8B	0.9700	C6—H6	0.9300
O1—C1	1.418 (2)	C5—C4	1.368 (4)
O1—H101	0.8200	C5—H5	0.9300
C9—C10	1.504 (3)	C11—H10A	0.9600
C9—C11	1.519 (3)	C11—H10B	0.9600
C9—H8	0.9800	C11—H10C	0.9600
C1—C2	1.509 (3)	C4—H4	0.9300
			
O3—N2—O2	121.46 (16)	C3—C2—C1	120.90 (18)
O3—N2—O4	120.26 (16)	C7—C2—C1	122.68 (17)
O2—N2—O4	118.27 (16)	C6—C7—C2	122.1 (2)
C8—N1—C9	114.86 (13)	C6—C7—Cl1	118.28 (17)
C8—N1—H2A	108.6	C2—C7—Cl1	119.66 (16)
C9—N1—H2A	108.6	C4—C3—C2	122.0 (2)
C8—N1—H2B	108.6	C4—C3—H3	119.0
C9—N1—H2B	108.6	C2—C3—H3	119.0
H2A—N1—H2B	107.5	C9—C10—H9A	109.5
N1—C8—C1	111.59 (14)	C9—C10—H9B	109.5
N1—C8—H8A	109.3	H9A—C10—H9B	109.5
C1—C8—H8A	109.3	C9—C10—H9C	109.5
N1—C8—H8B	109.3	H9A—C10—H9C	109.5
C1—C8—H8B	109.3	H9B—C10—H9C	109.5
H8A—C8—H8B	108.0	C5—C6—C7	119.7 (2)
C1—O1—H101	109.5	C5—C6—H6	120.2
N1—C9—C10	110.28 (16)	C7—C6—H6	120.2
N1—C9—C11	108.21 (16)	C6—C5—C4	119.8 (2)
C10—C9—C11	112.7 (2)	C6—C5—H5	120.1
N1—C9—H8	108.5	C4—C5—H5	120.1
C10—C9—H8	108.5	C9—C11—H10A	109.5
C11—C9—H8	108.5	C9—C11—H10B	109.5
O1—C1—C2	113.60 (15)	H10A—C11—H10B	109.5
O1—C1—C8	111.75 (15)	C9—C11—H10C	109.5
C2—C1—C8	108.90 (15)	H10A—C11—H10C	109.5
O1—C1—H1	107.4	H10B—C11—H10C	109.5
C2—C1—H1	107.4	C5—C4—C3	120.0 (2)
C8—C1—H1	107.4	C5—C4—H4	120.0
C3—C2—C7	116.40 (19)	C3—C4—H4	120.0
			
C9—N1—C8—C1	177.99 (14)	C1—C2—C7—C6	179.4 (2)
C8—N1—C9—C10	−68.4 (2)	C3—C2—C7—Cl1	−178.09 (18)
C8—N1—C9—C11	167.93 (18)	C1—C2—C7—Cl1	0.5 (3)
N1—C8—C1—O1	−59.77 (18)	C7—C2—C3—C4	0.0 (4)
N1—C8—C1—C2	173.93 (14)	C1—C2—C3—C4	−178.7 (2)
O1—C1—C2—C3	−44.5 (3)	C2—C7—C6—C5	−1.1 (4)
C8—C1—C2—C3	80.7 (2)	Cl1—C7—C6—C5	177.8 (2)
O1—C1—C2—C7	136.94 (19)	C7—C6—C5—C4	0.6 (4)
C8—C1—C2—C7	−97.8 (2)	C6—C5—C4—C3	0.2 (4)
C3—C2—C7—C6	0.8 (3)	C2—C3—C4—C5	−0.5 (4)

### Hydrogen-bond geometry (Å, °)

**Table:**

*D*—H···*A*	*D*—H	H···*A*	*D*···*A*	*D*—H···*A*
N1—H2A···O4	0.90	1.97	2.843 (2)	163
N1—H2B···O2^i^	0.90	1.93	2.8234 (19)	170
O1—H101···O4^ii^	0.82	1.98	2.7614 (19)	158

Symmetry codes: (i) −*x*+1/2, *y*+1/2, −*z*+1/2; (ii) −*x*+1, −*y*+1, −*z*+1.
